# Nalidixic acid—a good marker of fluoroquinolone resistance mechanisms in *Escherichia coli*

**DOI:** 10.1128/spectrum.01974-24

**Published:** 2025-05-22

**Authors:** Sofia Kalinen, Heini Kallio, Teemu Kallonen, Juha Knaapila, Tarja Lamminen, Pentti Huovinen, Peter Boström, Antti J. Hakanen, Marianne Gunell

**Affiliations:** 1 Institute of Biomedicine, University of Turku 169300, Turku, Finland; 2 Department of Clinical Microbiology, Turku Clinical Microbiome Bank, Turku University Hospital, Turku, Finland; 3 Department of Urology, Turku University Hospital, Turku, Finland; University of Saskatchewan, Saskatoon, Saskatchewan, Canada

**Keywords:** fluoroquinolone, drug resistance mechanisms, *Escherichia coli*, nalidixic acid, ciprofloxacin, pefloxacin

## Abstract

The purpose of this study was to evaluate how ciprofloxacin, pefloxacin, and nalidixic acid disks perform in screening fluoroquinolone resistance mechanisms in 278 *Escherichia coli* isolates collected from a prospective clinical material. Antimicrobial susceptibility testing of ciprofloxacin, pefloxacin, and nalidixic acid was performed with the disk diffusion method. PCR-based and sequencing methods were used to detect chromosomal mutations in the *gyrA* and *parC* genes and the presence of plasmid-mediated *qnr* and *aac(6′)-1b-cr* genes. In addition, whole-genome sequencing was used to confirm these results. Our results show that fluoroquinolone resistance mechanisms were discovered, even in ciprofloxacin-susceptible isolates, and plasmid-mediated low-level fluoroquinolone resistance is easily missed if only ciprofloxacin disk is used. *E. coli* strains with chromosomal *gyrA* and/or *parC* mutations were well detected with pefloxacin disk. However, nalidixic acid was a superior tool to detect and differentiate between low- (plasmid-mediated) and high-level (chromosomal mutations) fluoroquinolone resistance in *E. coli*. Thus, more clinical studies are needed to evaluate the clinical relevance of fluoroquinolone resistance mechanisms in enteric bacteria and pathogens that show potential but are not yet phenotypically fluoroquinolone-resistant.

**IMPORTANCE:**

We show in our clinical setting that fluoroquinolone resistance mechanisms are discovered, even among phenotypically fluoroquinolone-susceptible *Escherichia coli* isolates. When plasmid-mediated quinolone-resistance determinants are present, they are a potential risk for treatment failures due to accumulation of resistance mechanisms during the antimicrobial treatment. Therefore, when it is clinically relevant, fluoroquinolone resistance mechanisms in *E. coli* should be monitored more closely, and we also recommend testing nalidixic acid susceptibility.

## INTRODUCTION

Fluoroquinolones are widely used orally administered antimicrobials worldwide mainly due to their good efficiency against gram-negative bacteria. The extended use has increased fluoroquinolone resistance, which has also been linked to treatment failures (1). Low-level fluoroquinolone resistance is mainly caused by plasmid-mediated quinolone resistance (PMQR) determinants like *qnr* genes and *aac(6′)-1b-cr*, whereas high-level resistance is triggered by several chromosomal mutations in the quinolone resistance determining region (QRDR) of the DNA gyrase (*gyrA* or *gyrB*) and the DNA topoisomerase IV genes (*parC* or *parE*) (1–5). In *Escherichia coli* (*E. coli*), resistance to fluoroquinolones is highly associated with mutations in the *gyrA* gene (3).

Fluoroquinolone susceptibility testing has evolved during the years when new fluoroquinolone resistance mechanisms have been found. Nalidixic acid, a first-generation quinolone, was previously recommended by the Clinical and Laboratory Standards Institute for screening fluoroquinolone resistance in invasive *Salmonella* isolates (6). Formerly, *Salmonella enterica* (*S. enterica*) strains with reduced fluoroquinolone susceptibility used to be fully resistant to nalidixic acid, and we had, therefore, validated nalidixic acid as a screening test for reduced fluoroquinolone-susceptible *S. enterica* (7). Later, we showed that there is a population of *S. enterica*, which shows reduced susceptibility to ciprofloxacin but was susceptible to nalidixic acid, leading the use of the nalidixic acid screening test questionable (8, 9). Thereafter, since nalidixic acid screening tests were no longer valid in detecting PMQR genes, the use of ciprofloxacin screening was suggested (8, 10). Later, in 2014, European Committee on Antimicrobial Susceptibility Testing (EUCAST) recommended using pefloxacin for screening fluoroquinolone resistance in *S. enterica* since low-level fluoroquinolone resistance could not be reliably detected with ciprofloxacin disk alone (11, 12). In addition, in 2022, EUCAST recommended using pefloxacin disks for the detection of fluoroquinolone resistance mechanisms also in other *Enterobacterales*, including *E. coli* (13).

The aim of this study was to compare how ciprofloxacin, pefloxacin, and nalidixic acid disk diffusion method perform in detecting fluoroquinolone resistance mechanisms in a prospective clinical material of fecal *E. coli* isolates.

## MATERIALS AND METHODS

In total, 278 clinical *E. coli* isolates were investigated. *E. coli* isolates were cultured from fecal swab samples taken during the prostate biopsy from men participating in the multi-IMPROD sub-study [Improved Prostate Cancer Diagnosis—Combination of Magnetic Resonance Imaging Targeted Biopsies and Biomarkers Multi-Institutional Study (multi-IMPROD), NCT02241122] in Finland between March 2015 and May 2017 (14). Fecal swab samples were cultured on CHROMagar Orientation plates (BD Diagnostics, Heidelberg, Germany), and a 5 µg-ciprofloxacin disk (Oxoid, Ltd., Basingstoke, UK) was placed on top of the culture to select the patient’s most resistant *E. coli* strain. After the overnight incubation, two to three bacterial colonies with *E. coli* morphology (mauve to light purple colonies) were selected preferably near the ciprofloxacin disk, and pure cultures were made from these on CLED plates (BD Diagnostics, Heidelberg, Germany). Matrix-assisted laser desorption/ionization time-of-flight (Bruker Daltonics, Bremen, Germany) was used for the species identification of the isolated strains, and only *E. coli* isolates, one per patient, were studied further.

Antimicrobial susceptibility testing (AST) was performed with the disk diffusion method according to the EUCAST guidelines for the following antimicrobials: ciprofloxacin (5 µg), pefloxacin (5 µg), and nalidixic acid (30 µg) (Oxoid, Ltd., Basingstoke, UK). *E. coli* ATCC 25922 was used as a control strain in AST. Antimicrobial susceptibility profiles of *E. coli* strains were determined according to EUCAST standard version 11 (15). EUCAST has no clinical breakpoints for nalidixic acid, but the ecological cut-off value (ECOFF) for *E. coli* is set to 19 mm.

Fluoroquinolone resistance mechanisms were studied in all *E. coli* isolates with ciprofloxacin inhibition zone ≤ 30 mm. Mutations in *gyrA* and *parC* genes were studied in ciprofloxacin-resistant strains (inhibition zone < 24 mm) and strains with nalidixic acid below ECOFF (6–18 mm).

Oligonucleotides gyrA_307_f 5′-AAGCCGGTACACCGTCGCGTACTT-3′ and gyrA_570_r 5′-TTTCGCCAGACGGATTTCCG-3′ were used to amplify a 263 bp fragment of the *gyrA* gene (8), and oligonucleotides parC_172_f 5′-GTCTGAACTGGGCCTGAATGC-3′ and parC_321_r 5′-AGCGGATAACGGTAAGAGAACGG-3′ were used to amplify a 149 bp fragment of the *parC* gene. The *gyrA* and *parC* PCR reaction (50 µL) contained 0.2 pmol/µL of each primer, 0.03 U/µL AmpliTaq Gold DNA polymerase, 5 µL AmpliTaq Gold buffer, 2 mM MgCl_2_, and 0.2 mM dNTP mix (Thermo Fisher Scientific Baltics, UAB, Lithuania). The reaction was amplified using the following protocol: initial denaturation at 94°C for 10 min following 37 cycles of 30 s at 94°C, 30 s at 55°C, and 90 s at 72°C. PCR products were purified with Exonuclease I- and FastAp Thermosensitive alkaline phosphatase enzymes (Thermo Fisher Scientific Baltics, UAB, Lithuania) and sequenced (BigDye v.3.1 sequencing using ABI3730xl DNA analyzer, Institute for Molecular Medicine in Finland, FIMM, Helsinki, Finland) to reveal mutations in *gyrA* and *parC* QRDRs.

Transferable PMQR genes, *qnr* and *aac(6′)-1b-cr*, were screened for all the *E. coli* isolates with a ciprofloxacin disk inhibition zone ≤ 30 mm. In addition, 34 out of 90 randomly selected isolates with a ciprofloxacin disk inhibition zone > 30 mm were screened for PMQR genes with previously reported primers and protocols (16, 17).

Whole-genome sequencing (WGS) was used to detect *gyrB* and *parE* mutations in randomly selected 27 *E. coli* isolates showing ciprofloxacin disk inhibition zone 6 to 29 mm and confirm resistance mechanisms detected with conventional methods. Total genomic DNA of *E. coli* bacterial culture was extracted using the NucleoSpin Microbial DNA Kit (Macherey-Nagel, Germany). Library preparation was performed with Nextera XT (Illumina, USA) and sequencing with Illumina Nextseq2000 using P1 300-Cycle Kit (Illumina, USA). Fastq files were analyzed as paired, trimmed pairs with CLC Genomic Workbench Microbial Module version 25.0 (QIAGEN, USA). Resistance genes were screened using Find Resistance with PointFinder 1.2, PointFinder database for *E. coli* (3.0.1), and resistance genes were analyzed using Find Resistance with Nucleotide Database 1.3. Sensitivity and specificity of the tested antibiotic disks to detect fluoroquinolone resistance mechanisms were determined using MedCalc software (18).

## RESULTS

**Fig 1:**
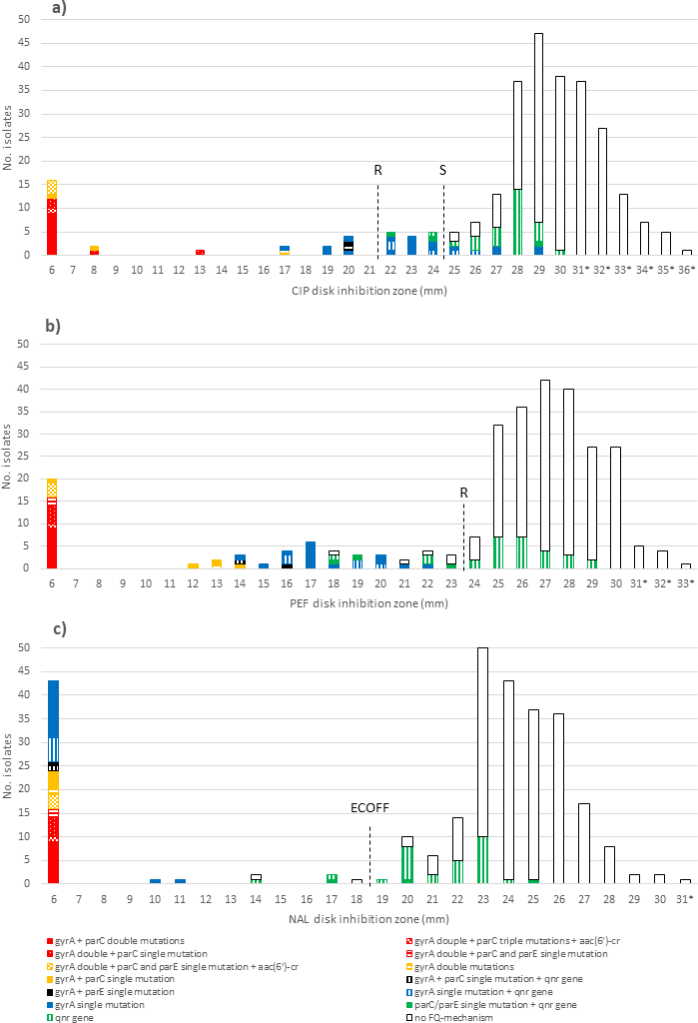
Ciprofloxacin (CIP, 1a), pefloxacin (PEF, 1b) and nalidixic acid (NAL, 1c) disk inhibition zone correlation to fluoroquinolone resistance mechanisms. Black dashed line in Fig. 1a and b represents the EUCAST resistance (R) and susceptible (S) breakpoint and ECOFF in Fig. 1c. *Randomly selected 34 out of 90 *E. coli* strains with a ciprofloxacin inhibition zone > 30 mm were screened for PMQR genes with PCR.

Of the 278 *E. coli* isolates, 27 (9.7%) were ciprofloxacin-resistant (CIP-R, inhibition zone < 22 mm); 14 isolates (5.0%) were susceptible, increased exposure (CIP-I, inhibition zone 22–24 mm); and 237 isolates (85.3%) were ciprofloxacin-susceptible (CIP-S, inhibition zone > 24 mm). Pefloxacin resistance (PEF inhibition zone < 24 mm) was detected in 56 *E. coli* isolates (20.1%), and 45 isolates (16.2%) showed high-level resistance to nalidixic acid (NAL inhibition zone 6–11 mm), and 51 isolates were below ECOFF value (<19 mm) (Fig. 1). Among the high-level nalidixic acid-resistant strains, the pefloxacin disk inhibition zone varied from 6 to 22 mm (i.e., strains were also pefloxacin-resistant), whereas the ciprofloxacin disk inhibition zone varied between 6 and 29 mm. Of the 56 pefloxacin-resistant strains, 27 were ciprofloxacin-resistant; 13 were ciprofloxacin-susceptible, increased exposure; and 16 isolates were ciprofloxacin-susceptible. Of the pefloxacin-resistant isolates, the nalidixic acid inhibition zone varied from 6 to 25 mm. Distribution of ciprofloxacin, pefloxacin, and nalidixic acid disk inhibition zones and their correlations to fluoroquinolone resistance mechanisms are presented in Table 1 and Fig. 1.

**TABLE 1:** Correlation of ciprofloxacin (CIP), pefloxacin (PEF), and nalidixic acid (NAL) disk inhibition zone (mm) to fluoroquinolone (FQ) resistance mechanism

FQ resistance mechanism				Strains	Disk inhibition zone (mm)		
gyrA	parC	parE	PMQR	n	CIP	PEF	NAL
S83L + D87N	S80I + E84V/G	nd	-	*9*	6	6	6
S83L + D87N	S80I + E84V + I259L	nd	*aac(6′)-cr*	*1*	6	6	6
S83L + D87N	S80I	nd		*4*	6–13	6	6
S83L + D87N	S80I	S458A/L416F	*aac(6')-cr*	*3*	6	6	6
S83L + D87N	S80I	L416F	-	2	6–8	6	6
S83L + D87N	-	nd	*-*	*1*	17	13	6
S83L	S80I	nd	*-*	*2*	19	12–14	6
S83L	E84G	nd	*qnr*	*1*	20	14	6
S83L	E84G	nd	*-*	*2*	20–22	6–13	6
S83L	-	I529L	*-*	1	20	16	6
S83L	-	nd	*qnr*	*5*	6–26	16–20	6
S83L	-	nd	*-*	*12*	17–29	14–22	6–10
D87T	-	nd	*-*	*1*	22	15	6
D87N	-	nd	*-*	*1*	27	20	11
-	-	nd	*qnr*	*28*	24–30	18–29	14–24

^*a*^ nd = not determined, - = negative for tested resistance mechanisms.

Fluoroquinolone resistance mechanisms were found in all 41 ciprofloxacin-resistant or -susceptible, increased exposure *E. coli* strains (i.e., CIP disk inhibition zone < 25 mm). Of the 27 ciprofloxacin-resistant strains, 23 had point mutations in the QRDR of both *gyrA* and *parC*, and four had point mutations only in *gyrA*. WGS analysis showed that five high-level ciprofloxacin-resistant strains with double *gyrA* mutation and single *parC* mutation also had mutations in *parE*, and three of these had also *aac(6′)-Ib-cr*. In addition, one strain with a single *gyrA* mutation also had a *parE* mutation and *qnr* gene. Of the 14 ciprofloxacin-susceptible, increased exposure *E. coli* strains, one had both *gyrA* and *parC* mutations, and 10 had *gyrA* mutations, of which three also had the *qnr* gene. One strain had a *parC* mutation with *qnr*; one had only *qnr*; and one had a *parE* mutation with *qnr* gene based on WGS analysis. However, single *gyrA* mutations were also found among ciprofloxacin-susceptible strains. A total of seven strains with ciprofloxacin inhibition zones between 25 and 29 mm had a single *gyrA* mutation, and two of them also had the *qnr* gene. In addition, 27 strains had the *qnr* gene (Table 1; Fig. 1a). One of the *qnr*-positive strains also had a *parE* mutation based on WGS analysis. Ciprofloxacin disk sensitivity to detect fluoroquinolone resistance mechanisms among our material was 53.95%, and specificity was 100.0%.

Of the 56 pefloxacin-resistant strains, 24 had point mutations in the QRDR of both *gyrA* and *parC*, and 21 had mutations only in the *gyrA* gene. WGS analysis revealed that five of these strains with double *gyrA* and single *parC* mutation also had a mutation in *parE*, and three of these strains also had *aac(6′)-Ib-cr* gene. One strain had a mutation in *parC* with the *qnr* gene. Two strains had a mutation in *parE*, and both of them also had the *qnr* gene. Ten strains did not have any QRDR mutations in *gyrA* or *parC*; five of them had only *qnr* genes; and two of them had also *parE* mutation. Five strains remained negative for all tested fluoroquinolone resistance determinants (Table 1; Fig. 1b). In addition, *qnr* genes were found in 25 pefloxacin-susceptible *E. coli* strains. Pefloxacin disk sensitivity to detect fluoroquinolone resistance mechanisms among our material was 67.11%, and specificity was 97.51%.

Of the 45 strains with nalidixic acid inhibition zone 6–11 mm, all had chromosomal mutations in a QRDR. Mutations in both *gyrA* and *parC* genes were detected in 24 strains, and 21 strains had mutations only in the *gyrA* gene. WGS analysis revealed that five of the strains with double *gyrA* and single *parC* mutation also had *parE* mutation, and three of these had also *aac(6′)-Ib-cr* gene. In addition, one strain with a *gyrA* single mutation also had a *parE* mutation. Combinations of different *gyrA* and *parC* mutations found in *E. coli* strains are presented in Table 1. Of the five strains with nalidixic acid inhibition zone 14–18 mm (i.e., below ECOFF value), one had a *parC* mutation with *qnr* gene; two had only *qnr* genes; and two strains remained negative for all fluoroquinolone resistance mechanisms. However, PMQR genes were detected in 28 strains with nalidixic acid inhibition zone up to 25 mm (Table 1; Fig. 1c), and WGS analysis showed that two of them had also *parE* mutations. Nalidixic acid disk sensitivity to detect fluoroquinolone resistance mechanisms below ECOFF value in our material was 63.16%, and specificity was 99.00%. The frequency of detected PMQR genes in our study population was 5.8% (16 strains), 4.0% (11), 3.6% (10), and 1.4% (4) for *qnrA*, *qnrB*, *qnrS*, and *aac(6′)-1b-cr*, respectively. Among 28 *E. coli* strains, a PMQR gene was the only resistance determinant found (Table 1).

## DISCUSSION

It has been previously shown that phenotypic ciprofloxacin susceptibility correlates poorly with genotypic resistance in *Enterobacterales* (19), and our data support this finding. We have shown in this study that based on EUCAST breakpoints (15), fluoroquinolone resistance mechanisms are found both in ciprofloxacin-resistant and -susceptible *E. coli* strains isolated from clinical fecal swab samples. Double *gyrA + parC* mutations were only detected in high-level ciprofloxacin-resistant isolates, whereas single *gyrA* mutations were detected, even in phenotypically ciprofloxacin-susceptible isolates. Thus, current breakpoints for phenotypic ciprofloxacin susceptibility do not correlate to acquired resistance mechanisms.

Pefloxacin is recommended for screening fluoroquinolone resistance mechanisms in *S. enterica* and other *Enterobacterales* (11, 13). In addition, it has been previously suggested that it would be beneficial to use both pefloxacin and nalidixic acid for screening low-level fluoroquinolone resistance in *E. coli*, and pefloxacin could perform better for detecting PMQR genes (20). Our results are in line with these findings. However, in our material, pefloxacin disk diffusion test differentiated well isolates with chromosomal mutations in QRDR of *gyrA* or *parC* since chromosomal mutations were detected only in phenotypically pefloxacin-resistant *E. coli* strains. While in our clinical material, PMQR genes were found in strains with pefloxacin inhibition zone up to 30 mm (i.e., pefloxacin-susceptible strains) and, conversely, in some pefloxacin-resistant strains, PMQR genes were the only resistance mechanisms found. Thus, clinical breakpoints for pefloxacin resistance correlate well with chromosomal mutations but inadequately with PMQR genes. The discrepancy between these results could be due to study material: all of our *E. coli* isolates were collected from fecal swab samples, whereas Dellgren et al. (20) have used both blood culture-positive, urine culture-positive, and fecal *E. coli* isolates and an additional collection of isolates with PMQR genes.

Commonly, double or triple mutations in both QRDR of *gyrA* and *parC* are needed for bacteria to become high-level fluoroquinolone-resistant (1–5). However, even one mutation in a QRDR of *gyrA*, such as S83L mutation in *gyrA*, S80I in *parC*, or D87N in *gyrA*, can lead to clinically relevant fluoroquinolone resistance in *E. coli* (21). In the present study, *gyrA* mutations were found in all phenotypically high-level fluoroquinolone-resistant isolates. This was in concordance with previous findings showing that high-level fluoroquinolone resistance is highly uncommon without S83L or D87N mutations in *gyrA* (3, 10). In the present study, WGS was used to screen *gyrB* and *parE* mutations in the selected isolates with ciprofloxacin disk inhibition zone 6–29 mm. Since no *gyrB* mutations were found, and only eight *parE* mutations were detected, of which only two were found in strains with no other gyrase or topoisomerase mutations, we can conclude that mutations in *gyrA* and *parC* are the most relevant fluoroquinolone resistance mechanisms for *E. coli*.

PMQR determinants like *qnr* genes are linked to low-level fluoroquinolone resistance and also enhance the selection of high-level resistance. Unfortunately, *qnr* genes are easily missed until further mechanisms are acquired and detected (2, 22, 23). We have previously shown a good correlation between high-level nalidixic acid resistance and ciprofloxacin resistance among *Salmonella* isolates (7). Later, we showed that low-level ciprofloxacin-resistant *S. enterica* are nalidixic acid-susceptible, and this low-level resistance is caused by PMQR genes (8, 9). In the present study, we have shown that among clinical *E. coli* isolates, the nalidixic acid disk diffusion test differentiated well between strains with chromosomal gyrase mutations and strains with PMQR genes, but only when high-level resistance was taken into account. In our material, the ECOFF value did not differentiate between isolates with PMQR genes and isolates with mutations in a QRDR of *gyrA* or *parC*. However, *gyrA* mutations were found only in phenotypically high-level nalidixic acid-resistant strains (inhibition zone 6–11 mm), as previously published (5, 8, 10). One *parC* mutation with the *qnr* gene was detected in strains with a nalidixic acid inhibition zone of 17 mm and two *parE* mutations in strains above nalidixic acid ECOFF. Otherwise, only *qnr* genes were detected in strains with nalidixic acid disk inhibition zones > 11 mm. It is of note that the number of strains carrying *aac(6′)-1b-cr* was low in our clinical material. However, 27 strains with nalidixic acid disk inhibition zone 6–27 mm were analyzed with WGS, indicating that *aac(6′)-1b-cr* had no influence on nalidixic acid disk specificity.

It is of note that there are also other fluoroquinolone-resistance mechanisms that were not analyzed in this study. For instance, low-level resistance causing quinolone efflux pump genes *qepA*, *oqxAB*, and *CrpP* (9, 24) and mutations in the other DNA gyrase and DNA topoisomerase IV genes *gyrB* and *parE* were screened only in 27 *E. coli* isolates. The selection of tested resistance genes may have had an impact on our results. However, the significance of *gyrB* and *parE* for fluoroquinolone resistance in clinical bacterial isolates is very minor (5). Five pefloxacin-resistant isolates and two isolates with nalidixic acid inhibition zone below ECOFF, without resistance mechanisms, were found among the study population. These strains were analyzed with WGS, and no additional mechanisms were detected. Thus, we can conclude that the significance of these other mechanisms in our study material was not significant.

The basis for AST is to differentiate between phenotypically resistant bacterial isolates having acquired resistance mechanisms and susceptible isolates that do not have acquired resistance mechanisms. If the bacteria have acquired a resistance mechanism but remain phenotypically susceptible, or susceptible, and you increase exposure (i.e., I-area), can it be determined as clinically susceptible? Since the accumulation of resistance determinants easily occurs once the first resistance mechanism has been acquired, even low-level fluoroquinolone resistance caused by PMQR genes can be a risk for treatment failures (3, 22). In this clinical *E. coli* material, PMQR genes were found among the phenotypically ciprofloxacin-susceptible strains, which confirms the previous findings that *qnr* genes do not necessarily have clinical relevance without additional resistance mechanisms (22, 23). Although the antimicrobial treatment of *E. coli* strains with ciprofloxacin disk inhibition zone within I-area (susceptible, increased exposure) might succeed with higher doses of fluoroquinolone (14), it can be misleading to interpret these strains as susceptible. However, it was reassuring that among the evidently ciprofloxacin-susceptible *E. coli* isolates having disk inhibition zone 31–36 mm, no fluoroquinolone resistance determinants were found.

### Conclusions

This study shows that fluoroquinolone resistance mechanisms among the clinical fecal *E. coli* are found, even in ciprofloxacin-susceptible isolates. Therefore, plasmid-mediated and even chromosomal mutations causing low-level fluoroquinolone resistance are easily missed if only ciprofloxacin breakpoint is used. *E. coli* strains with chromosomal *gyrA* and/or *parC* mutations are well detected with the pefloxacin breakpoint. However, nalidixic acid is a superior antimicrobial to detect and differentiate between low- (PMQR) and high-level (chromosomal mutations in QRDR) fluoroquinolone resistance in *E. coli*. We conclude that more clinical studies are needed to show whether mechanisms causing low-level fluoroquinolone resistance in *E. coli* are clinically relevant. Nonetheless, these isolates are at a potential risk of developing high-level resistance; thus, this phenomenon should be epidemiologically monitored.

## Data Availability

The WGS data of selected 27 *E. coli* strains have been deposited in GenBank under accession number https://www.ncbi.nlm.nih.gov/bioproject/PRJNA1249384
